# Supplementary material for: Dolutegravir: Virologic response and tolerability of initial antiretroviral regimens for adults living with HIV
Source: PLoS One. 2020 Aug 31;15(8):e0238052. doi: 10.1371/journal.pone.0238052 (PMC7458288; doi:10.1371/journal.pone.0238052)
Supplement: Attachment — Submitted filename: Replay from authors to reviewers.docx [file pone.0238052.s002.docx]

**Manuscript PONE-D-19-33515**

**Title** - "DOLUTEGRAVIR: VIROLOGIC RESPONSE AND TOLERABILITY OF INITIAL ANTIRETROVIRAL REGIMENS FOR ADULTS LIVING WITH HIV "

Dear editor of PLOS ONE and reviewers of our manuscript,

Thank you for the thoughtful review of our article. In response to the editorial feedback, we have made the following substantive changes to the manuscript:

**REPLY FROM AUTHORS**

**Reviewer's Responses to Questions**

**Reviewer #1:** Correa et al describe their experiences with DTG containing regimens in a patient cohort from Brazil. As highlighted by the authors, the data on efficacy and safety of DTG, also in real life settings, has been extensively studied. This limits the novelty of this manuscript apart from that it is from Brazil, a country where like in the main studies on DTG subtype B is dominant and where DTG has been introduced in Jan 2017. My main comment therefore is that the authors fail to make clear to me what their study and research adds to the already existing clinical information on DTG, both regarding wk24 data and tolerability. Without that, it would add little to the scientific community as lowlevel evidence retrospective analysis of a single center experience unfortunately.

**Other major comments:** The authors state that no real life data has been published. However, publications on this do exist. For example, there is a recent publication from Brasil which highlights the high potency of DTG in an analysis of 112243 patients who were prescribed DTG or one of to other regimens (Pascom AR JIAS 2019). Also, another study from Brazil on 107647 patients (including 10.5% on DTG) showed a very favorable suppression rate on DTG which was also comparable to the suppression rate in this study (89.1% vs 90.5%). It is unclear to me how the authors can substantiate their statement or how their study differs from these studies.

**Reply from authors: Brazil has different social and economic aspects between the regions of the country and within each region. These aspects affected the treatment response, treatment adherence, health support of patient by local health system, because this is important that local studies to be carried and the evidence compared with the national reality.**

Although it seems to be a retrospective analysis, the authors should state whether it was prospective or retrospective in their methods section.

**Reply from authors: The study was a retrospective cohort. Corrected in Materials and methods.**

The in- and exclusion criteria, data collection and the primary outcomes are not (well) defined.

**Reply from authors: Included the exclusion criteria and the primary outcome in the text and reformulated the description of data collection.**

The main reason to do the study was to 'aimed to estimate the frequency of individuals with a virologic response by week 24 of antiretroviral treatment and to describe the adverse events of the regimen containing dolutegravir'. In that perspective it is a main limitation that only from a approx 2/3 of total patients had lab data available. From the text it is unclear why the 93 patients were excluded (it reads '93 individuals provided had laboratory data'?)

**Reply from authors: For standardization purposes, all viral load and CD4 records of the study participants were collected following the hospital routine. Due to this, is expected not all patients had viral load and CD4 cell count measurements consistently. Of 230 patients, 93 were excluded for not having at least two available HIV viral load measurements collected. The data of adverse events was collected from patient records, only 8 patients did not have this data in their medical records.**

Open door, but 24 weeks of follow up is too short to make any meaningful conclusions in my perspective for a real life data study and this should be acknowledged in a better way than it is currently presented. Longer follow up is required. To present the limitations of the present study as 'some methodological limitations' is an understatement in my perspective and the authors should evaluate that more thoroughly.

**Reply from authors: We consider the main methodological limitations the period of follow-up of 24 weeks, that may have underestimated the effect of DTG regime, but it was possible to show that the proportion of virologic response corroborated with the literature findings, although the observation period was not long.**

**Minor comments:** What do the authors mean by 'bidirectional cohort study'?

**Reply from authors: The study was a retrospective cohort. Corrected in method.**

**Reviewer #2:** The manuscript by Correa A et al. is well written and structured. The work describes a real-life experience in the use of dolutegravir in a cohort of HIV infected patients. There are several data in literature describing the use of dolutegravir in clinical practice: I think that the Authors should improve their discussion comparing the data presented with these other studies. Moreover, it should be clarified if the adverse events during the treatment with dolutegravir led to the discontinuation of the regimen.

**Reply from authors: In our findings the discontinuation of the regimen occurred in only 9 of 222 participants (4.1%). Six of these patients were being treated for tuberculosis, disease with high prevalence in the studied region, and the other 3 cases was due to pregnancy, one individual with a psychiatric disease prior to initiating ART, and one had no information in the medical records.**

**Reviewer #3:** The present manuscript evaluated the efficacy and safety of a first line antiretroviral therapy based on dolutegravir. The authors concluded that dolutegravir based combination in first line therapy is a well tolerated and effective drug in the first 24 week follow-up. However, several aspects should be examined before consider it for publication. In more detail:

**Major comments:** 1. Patient population: A number of different patients have been used to assess toxicity and efficacy. This difference makes it difficult to interpret the results. - In the results section the authors stated that “Of the 562 individuals taking dolutegravir registered on SICLOM, we identified 230 who initiated the first antiretroviral regimen containing dolutegravir” However, only 222 were included in the tolerability analysis and 137 for the virological response. I suggest unifying the population for a better understanding of the results.

**Reply from authors: For standardization purposes, all viral load and CD4 records of the study participants were collected following the hospital routine. Due to this, is expected not all patients had viral load and CD4 cell count measurements consistently. Of 230 patients, 93 were exclude for don’t had at least two available HIV viral load measurements collected. The data of adverse events was collected from patient records, only 8 patients did not have this data in their medical records. We analyzed the adverse event with the 137 patients but we considerate that the discrepancy not produce selection bias.**

- Are there any baseline difference between the population included for tolerability and virological response?

| Characteristics | Not included in the virological response analysis  (n = 85) | Included in the virological response analysis  (n = 137) | p-value |
| --- | --- | --- | --- |
| Biological |  |  |  |
| Ageᵃ | 33,9 ± 10,2 | 34,8 ± 9,9 | 0,509 |
| Sex |  |  |  |
| Male | 71 (83.5%) | 90 (65.6%) | 0.004 |
| Habits |  |  |  |
| Use of illicit drugs | 8/44 (18.2%) | 18/107 (16.8%) | 0.841 |
| Alcohol consumption | 27/41 (65.8%) | 67/104 (64.4%) | 0.871 |
| Smoker | 9/44 (20.4) | 30/97 (30.9%) | 0.198 |
| Clinical characteristics related to HIV |  |  |  |
| Time between HIV diagnosis and initiating ART (months)ᵇ | 2.0 (0.8 – 3.0) | 2.0 (1.2 – 3.9) | 0.274 |
| Max. Viral load (copies/mm3)ᵇ | 25353  (5916 – 219618) | 36529  (7115 - 149156) | 0.878 |
| CD4 nadir (cell/mm3) ᵇ | 377.5 (177 – 528) | 377 (162 – 556) | 0.742 |
| Adverse event | 2 (2.3%) | 21 (15.3%) |  |

ᵇ Median (P25 - P75)

**Reply from authors: Comparing the characteristic of patients, the frequency of males shows statistic difference. We believe that this difference not modify the estimate of virological response, the association with virological response was not significant in this study (p = 0.623 – table 3).**

- How many patients included in the virological study had toxicity events? What are the efficacy and safety data for the same population studied (f.e: 137)?

**Reply from authors: Of the 137 individuals included in the virological study, 21 had adverse event.**

2. Immunologic recover:

- Although the baseline value of CD4 of the study population is high, 392, it is noteworthy that the average recovery of CD4 at 24-36 weeks of follow-up is only 50 cells. Given that the virological efficacy is high, what is the explanation for this relatively low immune response?

**Reply from authors: Our results show an increase mean of 50 cells each month. We did correction in the text making clear that is a monthly growth mean rate. Considering 24 weeks the mean of CD4 count of population was 625 cell and in the 36 weeks was 700 cells, approximately. We conclude that the immune response was satisfactory.**

- The information recorded in figure 2 does not reflect the poor CD4 recovery mentioned along the test. - Figure 2 represents mean CD4 counts:

o In what population?

**Reply from authors: individuals after the first antiretroviral regimen containing Dolutegravir.**

o There is a significant increase of CD4 counts from 390 up to 715 at 12 months. However, after that there is a decrease from 715 to 550. What is the explanation for that? Please clarify the information recorded in figure 2.

**Reply from authors: Although CD4+ cell count is an important test for monitoring antiretroviral therapy effectiveness and guiding opportunistic infection prophylaxis, values may vary from one measurement to another, fluctuating with diurnal variation and ongoing infections, especially with higher CD4+ counts. Once CD4+ cell count is good, it requires less frequent monitoring and practioners should look at overall measurements. In this regard, the viral load is the most important parameter in treatment monitoring.**

3. Virologic failure: - For the virological analysis only 137 patients has been considered. Among this group the efficacy seems to be similar to that presented in other studies. However, have you been observed any difference when stratified patients according their baseline viral load (<100,000 vs >100,000 copies/mL)? - There is no resistance analysis for the 15 patient with virological failure. Could you provide any data of resistance?

**Reply from authors: Among the 15 patients with virological failure, six achieved virological control beyond 24 weeeks, it shoul be noted that all of them had baseline viral load >100,000 copies/mL. Despite the fact that antiretroviral regimens with dolutegravir facilitate adherence they do not guarantee it totally, adherence is multifactorial and varies within individuals over time. Even though convenience and tolerability of ART have improved, suboptimal adherence remains common (Bangsberg DR, Kroetz DL, Deeks SG.** [**Adherence-resistance relationships to combination HIV antiretroviral therapy.**](https://www.inpractice.com/Textbooks/HIV/Antiretroviral_Therapy/ch13_pt1_Adherence/Chapter-Pages/Page-1) **Curr HIV/AIDS Rep. 2007 May;4(2):65-72; Dorward J, Lessells R, Drain PK et al. Dolutegravir for first-line antiretroviral therapy in low-income and middle-income countries: uncertainties and opportunities for implementation and research. Lancet HIV. 2018 Jul; 5(7): e400–e404). We found that nine patients who did not achieve virological control had suboptimal adherence with irregular follow-up visits. Two of them had genotypic assays with no evidence of resistance mutations and one patient had no genotypic data since viral load was low (<1000 copies/mL).**

Minor comments:

- Page 5 line 90: “93 individuals provided had laboratory data….” You men 93 individual provided had not….?

**Reply from authors: The text was corrected in the manuscript.**

- Page 6 line 100: “the median time using ART was 13.2 months.” If the median time on dolutegravir was 13 months why was the virologic analysis only performed at week 24?

**Reply from authors: Some individuals were considerate the CD4 count and viral load after the investigation period with objective to describe the CD4 cells and load viral behavior in the maximum time.**

- Table 1. Please specify if data recorded in this table correspond to 230, 222 or 137 patients.

**Reply from authors: The text was corrected in the manuscript.**

Table 2. Percentages of adverse events are referred to the 23 individuals who presented any event. This is confusing since it gives the impression that the adverse events rates were very high.

**Reply from authors: The text was corrected in the manuscript.**

Page 10 line 173. “….. of ART of 334,000 copies was reduced to a median of 193….” This information s not recorded in the result section.

**Reply from authors: Included** **in the text of manuscript – “The median viral load of patients at the beginning of ART was 334,000 copies, after 24 weeks decrease to 193 copies”.**
